# Role of Periostin in Adhesion and Migration of Bone Remodeling Cells

**DOI:** 10.1371/journal.pone.0147837

**Published:** 2016-01-25

**Authors:** Teresa Cobo, Cristina G. Viloria, Laura Solares, Tania Fontanil, Elena González-Chamorro, Félix De Carlos, Juan Cobo, Santiago Cal, Alvaro J. Obaya

**Affiliations:** 1 Departamento de Cirugía y Especialidades Médico-Quirúrgicas, Facultad de Medicina, Universidad de Oviedo, Asturias, Spain; 2 Departamento de Biología Funcional, Facultad de Medicina, Universidad de Oviedo, Asturias, Spain; 3 Departamento de Bioquímica y Biología Molecular, Facultad de Medicina, Universidad de Oviedo, Asturias, Spain; 4 Instituto Universitario de Oncología (IUOPA), Facultad de Medicina, Universidad de Oviedo, Asturias, Spain; University of Oulu, FINLAND

## Abstract

Periostin is an extracellular matrix protein highly expressed in collagen-rich tissues subjected to continuous mechanical stress. Functionally, periostin is involved in tissue remodeling and its altered function is associated to numerous pathological processes. In orthodontics, periostin plays key roles in the maintenance of dental tissues and it is mainly expressed in those areas where tension or pressing forces are taking place. In this regard, high expression of periostin is essential to promote migration and proliferation of periodontal ligament fibroblasts. However little is known about the participation of periostin in migration and adhesion processes of bone remodeling cells. In this work we employ the mouse pre-osteoblastic MC3T3-E1 and the macrophage-like RAW 264.7 cell lines to overexpress periostin and perform different cell-based assays to study changes in cell behavior. Our data indicate that periostin overexpression not only increases adhesion capacity of MC3T3-E1 cells to different matrix proteins but also hampers their migratory capacity. Changes on RNA expression profile of MC3T3-E1 cells upon periostin overexpression have been also analyzed, highlighting the alteration of genes implicated in processes such as cell migration, adhesion or bone metabolism but not in bone differentiation. Overall, our work provides new evidence on the impact of periostin in osteoblasts physiology.

## Introduction

Periostin, also named osteoblast-specific factor 2, is an ECM protein belonging to the fascilin-1 family of proteins. It was firstly identified as an osteoblast specific factor using techniques of subtraction hybridization and differential screening [1]. Periostin is mainly expressed in the periosteum, periodontal ligament and in osteoblastic cells on the alveolar bone surface in adult tissues [2], and its expression is induced by TGF-β [1]. Following its identification, it was proposed that periostin was a component of the extracellular matrix with a structural function. However nowadays it is known that periostin also plays important roles in functions essential for the maintenance of the normal activity of connective tissues. In fact, periostin is a 90 kDa secreted protein showing a complex structure composed of an amino-terminal EMI domain, a tandem repeat of 4 fas I domains, and a carboxy-terminal domain including a heparin-binding site [2–3]. After being secreted, the EMI domain, a small module rich in cysteine residues, is important to interact with type I collagen, fibronectin and Notch1; whereas the fas 1 domains interact with tenascin-C and BMP-1 [4–6]. Moreover, periostin is also able to establish interactions with αvβ3 and αvβ5 integrins, which underlines the importance of periostin in cell migration; and with laminin γ2, although the functional relevance of this interaction is still unknown [3, 7]. These interactions illustrate that periostin not only provides physical support but also regulates different aspects concerning to the differentiation, function or morphology of connective tissues.

Periostin has also been related to different pathological situations. Apart from its role in cell adhesion in bone physiology [1], periostin is required to adapt bone mass and ECM architecture in response to mechanical loading [5, 8–10]. Moreover, mouse lacking periostin show defects like dwarfism [9, 11] and, periostin expression has been detected in fibrous dysplasia, a benign bone disease [12]. In relation to tumorigenesis, high levels of periostin have been described in lung carcinoma (NSCLC), breast cancer, head and neck cancer, ovarian cancer or pancreatic ductal adenocarcinoma [13]. Of note, periostin participates in tumor development promoting cellular adhesion and enforcing tumor cell motility throughout the interaction with integrins αγβ3 and αγβ5 [14]. Different reports have also shown that high expression levels of periostin correlate with an increase of angiogenesis or metastasis [15–16].

During development, periostin is required for cardiovascular differentiation of cardiac valves and heart skeleton and, in general, presence of periostin has a beneficial effect in cardiovascular physiology [17–18]. For instance, periostin is expressed following myocardial injury [19], participating in bone marrow cells differentiation into cardiac fibroblasts, and further mobilization and tissue engraftment [20]. In allergic processes, periostin expression is stimulated by type-2 inflammatory cytokines [21–22]. In addition, in airway allergic reactions, periostin deposition may function to guide and facilitate granulocyte infiltration and to sustain inflammation [23]. High expression of periostin has also been described during cutaneous wound repair. In fact, increased levels of periostin are observed in the granulation tissues beneath wound edges and at dermal-epidermal junctions in wounded mice [24–25]. Furthermore, absence of periostin in knock-out mice compromises wound repair and re-epithelialization processes *in vivo* and impairs dermal fibroblasts proliferation and migration *in vitro* [24, 26].

Importance of periostin in oral health is underlined by the fact that, in adult tissues, it is expressed in periodontal ligament fibroblasts and in alveolar bone [27]. During embryogenesis, periostin can be detected in developing teeth at sites of epithelial-mesenchymal interaction suggesting a role in ECM organization [11]. Moreover, periostin-deficient mice show a wider periodontal ligament tissue, an inflammatory phenotype with a neutrophil infiltrate, enamel and dentin matrix defects as well as abnormal organization of alveolar bone, all of it resulting in a teeth unstable structure [11]. Periostin is also able to modulate expression of multiple downstream genes including α-smooth muscle actin (αSMA), collagen, fibronectin, aggrecan, sclerotin, chemokines, and TFG-β1 [9, 19, 28–29]. Most of the studies have been focused on the role of periostin in fibroblasts and especially in those fibroblasts of the periodontal ligament and their participation in tissue repair and recovery. However, periostin role in preosteoblast and odontoblasts is not fully understood. In this regard, it is known that participates in controlling postnatal tooth formation [27], and that its presence influences the differentiation process and stimulates cell recruitment and adhesion [9]. As an approach to unravel the role of periostin in bone physiology, we employ two different murine cell lines, MC3T3-1, a good model to study osteoblast differentiation; and RAW 264.7, a macrophage-like cell line, to overexpress exogenous periostin and to evaluate changes in cell behavior. Overall, our data suggest that high levels of periostin increase adhesion of these cells lines to different ECM components and reduce their migratory capacities. By contrast, downregulation of periostin by RNA interference considerable reduces the attachment of MC3T3-E1 cells to both type-1 collagen and fibronectin. We also performed RNA hybridization to evaluate changes at the genomic level following periostin overexpression. This analysis allowed us to establish novel associations between periostin and expression of genes related to cell adhesion, migration or ECM remodeling processes. These results may help to shed light on the participation of periostin in the physiology of osteoblasts.

## Materials and Methods

### Cell lines and transfection

MC3T3-E1 cell line (kindly provided by Dr. J.M. Ramis “Universitat de les Illes Balears”, Spain) was routinely maintained in MEM-α (supplemented with L-Glutamine, ribonucleosides and desoxirribinucleosides but not with ascorbic acid) medium and RAW 264.7 cell line (kindly provided by Dr. C. López-Otín “Universidad de Oviedo”, Spain) in RPMI 1640 GlutaMAX mediurm. In both cases, basal media was supplemented with 10% heat-inactivated foetal bovine serum and 100 U/mL penicillin and 50 μg/mL streptomycin. Cells were incubated at 37°C in a 5% CO_2_ supplemented atmosphere. Periostin cDNA (Origene MR210633) or for a mix of 4 x 29 mer shRNAs that specifically target POSTN RNAm in PGFP-v-RS vector (Origene TG509663) were transfected using TransIT-X2TM Dynamic Delivery System (Mirus) in OptiMEM I Reduced-Serum Medium (Invitrogen), following manufacturer instructions. To select stable clones geneticin was added to the medium at 500 μg/mL for MC3T3-E1 cells and 200 μg/mL for RAW 264.7 cells.

### Western-blot, inmunoprecipitation and inmunostaining

For western blot analysis, proteins were resolved by 8 or 12% polyacrylamide gel electrophoresis, transferred to a nitrocellulose membrane and subsequently probed with the indicated antibodies. Membranes were blocked using TBS-T (Tris-HCl 25mM pH 7.5, 150mM NaCl, 0.05% Tween-20) buffer including 5% non-fat dry milk (Biorad). The following primary antibodies were used: anti-periostin (Santa Cruz Biotechnology sc-67233), anti-p2rx7 (Santa Cruz Biotech, sc-31499), anti-IGFBP-5 (Santa Cruz Biotech, sc-6006), anti-LIFR (Santa Cruz Biotech, sc-659), anti-ADAM23 (Biorbyt), anti-p-Erk, anti-Erk, anti-p-Akt and anti-Akt (Cell Signalling Technology) and anti-DMP-1 (Ray Biotech). Immunoreactive proteins were visualized using HRP-peroxidase labeled secondary antibody and the ECL Luminata RM Forte Western HPR substrate. For extracellular periostin detection, cells were incubated in conditioned media (basal media without serum) for 16h. Recombinant periostin was inmunoprecipitated with anti-periostin antibody bound to gammabind G sepharose beads (Amersham). After extensive washing, inmunoprecipitated proteins were resolved by SDS-polyacrylamide gel electrophoresis and recombinant periostin was detected by western-blot using an anti-myc antibody (9E10, Santa Cruz Biotech). Media was also concentrated under speed vac and periostin presence detected by western-blot. To perform cell staining, MC3T3-E1 cells stably expressing periostin, or control cells carrying an empty vector, were fixed with 4% paraformaldehyde in phosphate-buffered saline. Samples were blocked with 15% foetal bovine serum in the same buffer. To detect recombinant periostin, blocked slides were incubated for 2 hours with the primary antibody against myc (9E10, Santa Cruz Biotech), followed by 2 hours of incubation with a secondary Alexa488-conjugated sheep anti-mouse antibody (GE Healthcare). In all samples, 4′,6′-diamino-2-phenylindole hydrochloride (DAPI) was added at 100 ng/ml to visualize DNA in the cell nucleus. Images were obtained using fluorescence microscopy and a digital camera.

### Adhesion assays

Adhesion assays were developed using a 96 well fluorimetric ECM Cell Adhesion Array Kit (Millipore) in triplicates for each cell culture condition, following instructions by the manufacturer. Briefly, 10^5^ cells were incubated for 2 h at 37°C. Then cells were lysed and data was obtained by fluorometry (485/530nm excitation/emission filters) with blank subtraction (adhesion on BSA) using a Synergy H4 Hybrid reader. All data are the mean of three independent experiments.

### Migration and proliferation assays

Migratory capacity of cells on the ECM components fibronectin, and type-I collagen was examined using IBIDI chambers in triplicates. Briefly, uncoated IBIDI dishes (ref #81151) were coated overnight at 4°C with type-1 collagen (Sigma-Aldrich) or fibronectin (Sigma-Aldrich). Then, a culture insert (IBIDI, ref #80206) was used to form chambers for cell seeding. After removal of the separation wall, cell migration was monitored under time lapse microscopy using Zeiss Axio Observer Microscopy. Migration distance of each cell line was quantified at different points (n = 6) using Image J. Results were obtained after 16 h migration for all the substrates. Cell migration was also monitored using standard tissue culture dishes suitable for wound healing migration assays (IBIDI, ref #81176) following manufacturer instructions. Proliferation rate was calculated by direct cell counting on four different fields over a 4 days period.

### RT-PCR analysis

Total RNA was isolated using TRIzol reagent (Invitrogen) by guanidium thiocyanatephenol-chloroform extraction and reverse transcription reactions were carried out with 300 ng of RNA, using the Thermoscript RT-PCR system (Invitrogen) with random hexamers. For analysis of periostin expression, 9 μl of a 1:5 dilution of cDNA was employed in quantitative PCR using the TaqMan probe Mm00450111_m1 and TaqMan Master Mix in an AbiPrism 7900HT (Applied Biosystems), and following manufacturers’ instructions.

### RNA expression arrays

Whole RNA from each cell line was isolated using TRIzol (Invitrogen) and purified with the RNeasy Mini Kit (Qiagen). Concentration and quality of samples were determined using an Agilent 2100 Bioanalyzer, and those with the best quality were selected for hybridization with a GC Mouse Gene 2.0 Array (Affymetrix), following the manufacturer’s instructions. Hybridization was performed at the “Centro de Investigación Médica Aplicada" (CIMA, Pamplona, Spain). Quality control of microarray data was performed using Affymetrix Expression Console. Data are expressed as base-2 exponents. Array data were deposited at the Gene Expression Omnibus with the accession number GSE66416. Bioinformatic analysis was performed using the Babelomics platform (http://www.babelomics.org/) and the Ingenuity Pathway Analysis platform from Quiagen.

### Statistical analysis

Statistical analysis were carried out using the GraphPad Prism 5.0 Software. Data are represented as means +/- S.E. The occurrence of significant differences was determined with the Student t test. p values under 0.05 were considered statistically significant (*p* < 0.05, *; *p* < 0.01, **; *p* < 0.005, ***).

## Results

### Generation of cell lines overexpressing periostin

**Fig 1:**
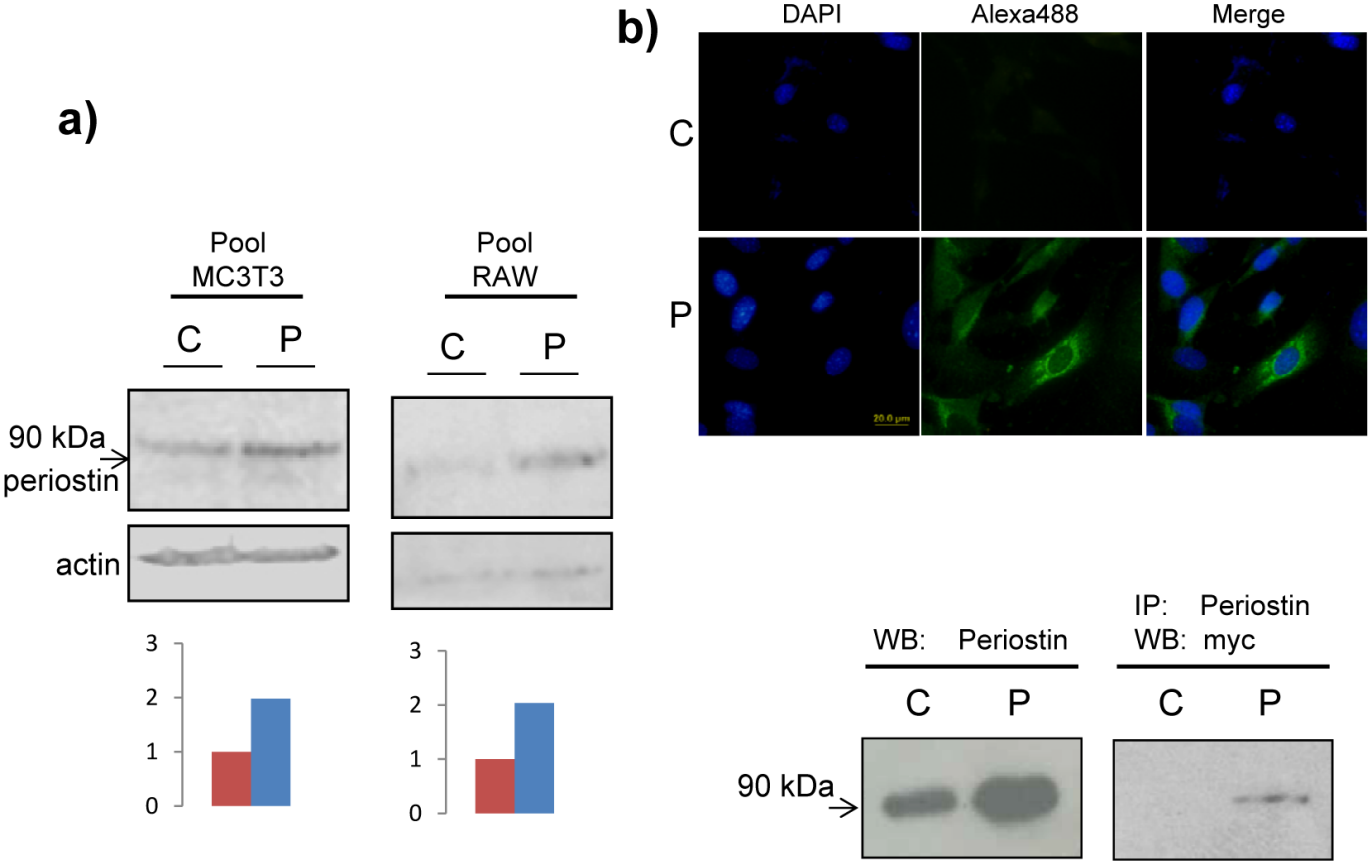
Selection of stable MC3T3-E1 and RAW 264.7 transfectants. a) Western blot analysis of MC3T3-E1 and RAW 264.7 producing exogenous periostin. Control, cells transfected with an empty vector. Top, detection with an anti-periostin antibody. An anti-Actin antibody was used as a loading control. Bottom, representation of normalized expression using values from Image J densitometry of western-blots. C, control cells. P, periostin overexpressing cells. b) Recombinant periostin is secreted to the cell medium. Top, Cellular inmunolocalization of periostin in MC3T3-E1 transfectants; cells using a specific anti-myc primary antibody and an Alexa-488-conjugated secondary antibody (green). DAPI staining was used to detect nuclei (blue). Bottom, recombinant periostin is detected either with anti-periostin or in an anti-periostin inmunoprecipitate with an anti-myc antibody by western-blot in MC3T3-E1 cells conditioned medium. C and P indicate conditioned medium of control cells transfected with an empty vector and with a vector containing the full-length cDNA for periostin tagged with a c-myc epitope respectively.

Although periostin effect on cell properties has already been described in several cell lines [30–33] we wanted to asses periostin effects in two murine cell lines directly implicated in bone—physiology and with antagonistic functions; the pre-osteoblastic MC3T3-E1 cell line, a good model for studying in vitro osteoblast differentiation; and the murine macrophage RAW264.7 cell line, with the capacity to differentiate to osteoclast-like cells. Both cell lines have been extensively used in order to unravel mechanisms towards osteoblastic and osteoclastic differentiation as well as to assay the effects of chemicals on bone physiology [34–42]. For that purpose, a vector containing periostin cDNA was used to transfect both cell lines and the presence of a immunoreative band at the expected size (90 kDa) was confirmed by western-blot analysis using an anti-periostin antibody (Fig 1a, top). An empty vector was employed as a control. Periostin 2-fold expression was estimated by image J quantification in cells transfected with cDNA for periostin respecting those cells transfected with an empty vector (Fig 1a, bottom). Localization of recombinant periostin exogenously expressed was observed in MC3T3-E1 cells transfected with a vector containing the full length cDNA for periostin. Inmunostainig was performed using an anti-myc antibody taking advantage of the myc epitope present in the recombinant periostin (Fig 1b, top). We also detected the recombinant protein in cell media either by western-blot and by inmunoprecipitation (Fig 1b, bottom). These data indicate that recombinant periostin is a secreted protein. Cells resulting from these transfection experiments were employed for further functional assays.

### Periostin increases cell adhesion to extracellular matrix components

**Fig 2:**
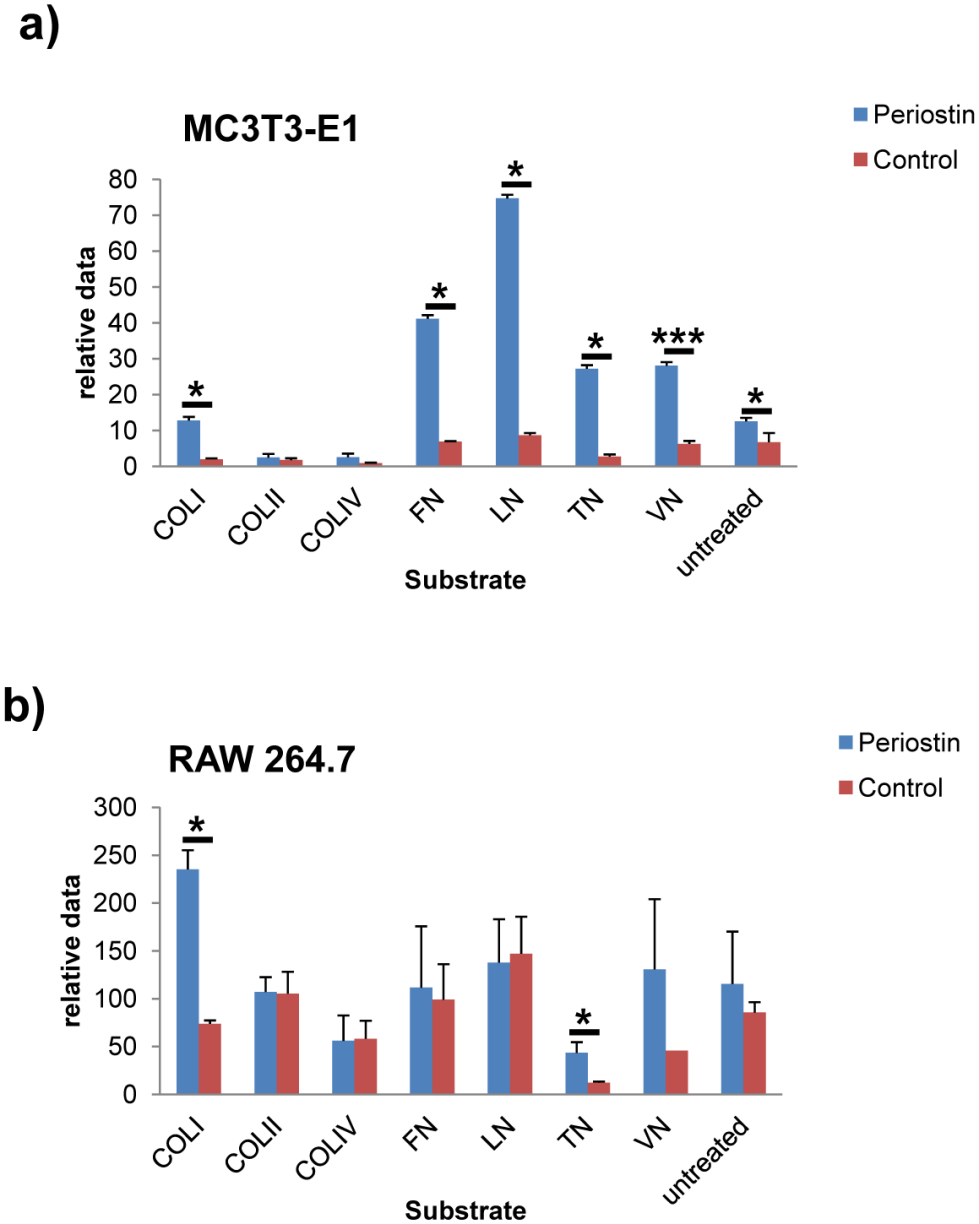
Adhesion profiles of MC3T3-E1 and RAW 264.7 stable transfectants on extracellular matrix proteins. Presence of periostin modifies the adhesion profile of MC3T3-E1 (a) and RAW 264.7 (b) cells to different ECM components. Col I, type I collagen; Col II, type II collagen; Col IV, type IV collagen; FN, fibronectin; LN, laminin; TN, tenascin; VN, vitronectin; untreated, standard culture dish with no specific coating. Y-axis, relative data refers to fluorometry (485/530nm excitation/emission filters) with blank subtraction (adhesion on BSA). Student t test (*p < 0*.*05*, *; *p < 0*.*01*, **; *p < 0*.*005*, ***).

Cell adhesion experiments were performed using the ECM Cell Adhesion Array Kit from Millipore. This array allowed us to examine the effect of periostin on cell binding to different extracellular matrix proteins such as collagens type-I, -II, -IV, fibronectin, laminin, tenascin and vitronectin. As it can be seen in Fig 2a, presence of periostin increases the capacity of MC3T3 cells to bind type-I collagen, fibronectin, laminin, tesnascin, vitronectin and untreated dish (normal culture dish) with respect to control cells. Thus, periostin overexpression is able to increase adhesion of MC3T3-E1 to these substrates by 6.38-fold, 5.94-fold, 8.62-fold, 9.87-fold, 4.46-fold and 1.85-fold respectively. In the case of RAW 264.7 cells, overexpression of periostin only increased the binding to ECM components such as type-I collagen and tenascin (3.18-fold and 3.56-fold compared to control cells, Fig 2b). For further experiments, we isolated individual clones of MC3T3-E1 transfectans and centered the following studies in a clone that showed 41-fold periostin overexpression over control cells, as assayed by qRT-PCR (clone P3). This clone P3 also showed differences in the adhesion profile to different ECM components, however, we did not detected alterations in growth rate when compared to control cells (S1 and S2 Figs). To examine whether downregulation of periostin in MC3T3-E1 cells could induce effects contrary to those observed by its overexpression, we carried out RNA interference by using gene specific shRNAs. Cells undergoing interference showed a considerable reduction in their adhesive properties towards type-1 collagen and fibronectin, the two ECM components mainly affected by the overexpression of periostin. This result reinforces the influence of periostin in the adhesion properties of MC3T3-E1 cells (S3 Fig).

### Periostin reduces migration capacity of MC3T3-E1 cells

**Fig 3:**
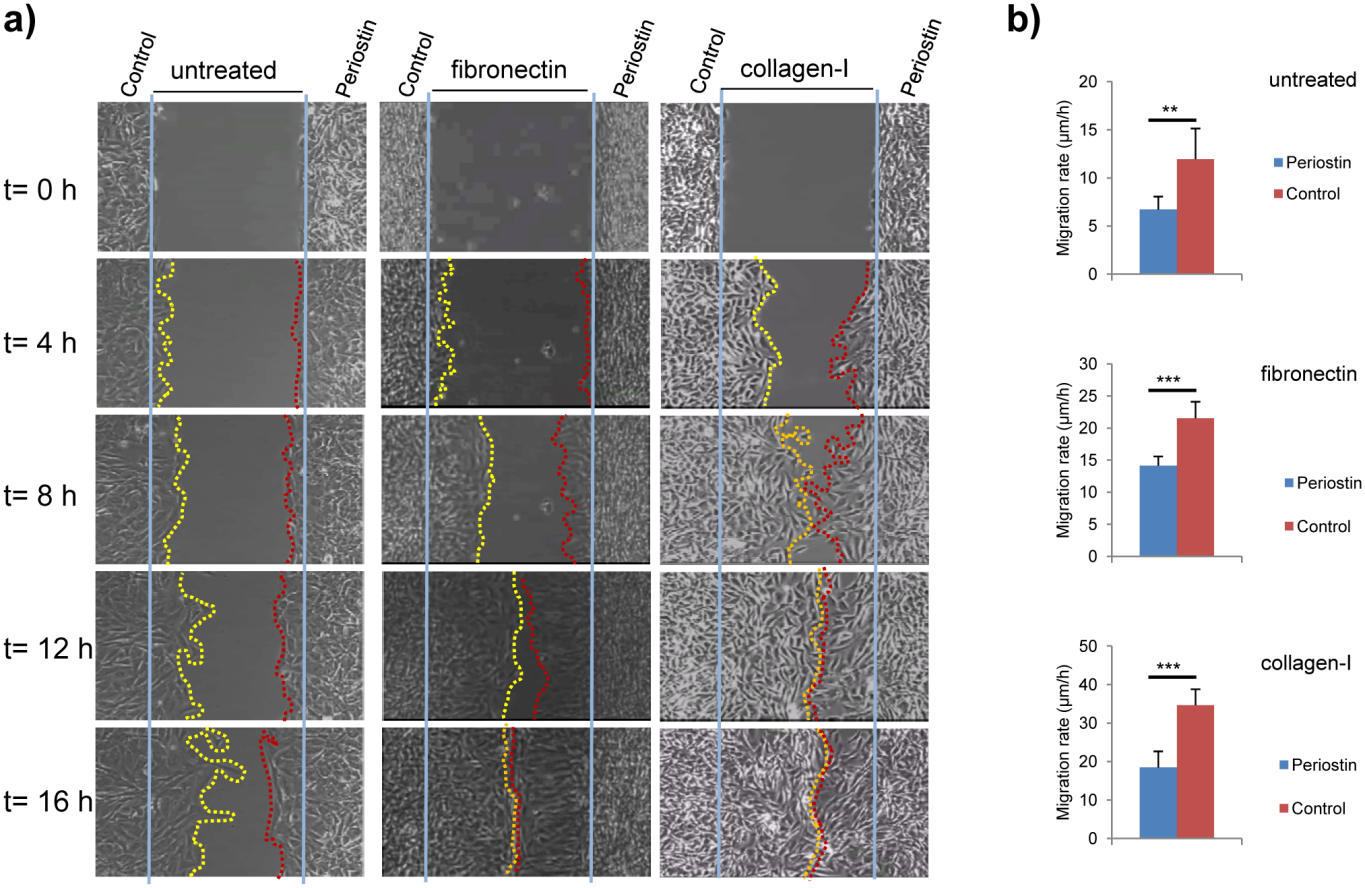
Periostin overexpressiong compromises migration properties of MC3T3-E1 cells. (a) MC3T3-E1 periostin-overexpressing (P) and control cells (C) were allowed to migrate simultaneously over a 500 μm gap in standard culture dishes commonly employed for wound healing assays (untreated) or wells coated with type-I collagen or fibronectin. Pictures at starting (t = 0 h), 4 h, 8 h, 12 h, and 16h time points are included. Starting point is indicated with a straight blue line and final points with a dotted line: yellow for MC3T3-E1 control cells and red for clone P3 cells. (b) Graphical representation of migration rate measured at 8 h from three independent experiments. Student t test (*p < 0*.*05*, *; *p < 0*.*01*, **; *p < 0*.*005*, ***).

Periostin is strongly up-regulated after tissue injury and is involved in early events of bone fracture repair, recruitment of progenitors, osteoblast differentiation and bone formation [10, 43]. With this in mind, we wanted to investigate whether periostin overexpression could affect MC3T3-E1 migratory abilities. To this end, we employed the Culture silicone inserts from IBIDI^®^ which are suitable for using upon different substrates. These inserts define gaps of 500 μm at the time of cell plating and migration can be monitored at different incubation times. Bearing in mind the previous results from the adhesion assays and particularly in the specific profile observed for the periostin overexpressing clone P3 (S1 Fig). To evaluate migration, we employed uncoated dishes that were overnight coated with either type-1 collagen or fibronectin. Results indicate that MC3T3-E1 cells overexpressing periostin showed a lower migratory capacity when they migrate on these two substrates (Fig 3a; S1 and S2 Movies). On fibronectin, MC3T3-E1 cells overexpressing periostin moved an average rate of 14.14 μm/h, whereas control cells moved at 21.52 μm/h (Fig 3b). In the case of type-I collagen-coated wells, average rate of MC3T3-E1 cells overexpressing periostin was 18.45 μm/h, and 34.62 μm/h in the case of control MC3T3-E1 cells (Fig 3b). Additionally, we also examined the migratory capacity of MC3T3-E1 cells using standard culture dishes commonly employed for wound healing assays (untreated) with the finding that MC3T3-E1 cells overexpressing periostin moved in an average rate of 3.19 μm/h over 8 h, which is significantly lower than the rate observed for control cells (6.72 μm/h)(Fig 3; S3 Movie). It is important to note that proliferation rate of control cell line and clone P3 is very similar with a doubling time of approximately 23h (S2 Fig). Taking together, these data suggest that presence of periostin could facilitate the attachment of bone remodeling cells to appropriate locations.

We also carried out similar experiments of migration and periostin interference on RAW264.7 transfectants (S4 Fig). Again, overexpression of periostin resulted in a lower migratory capacity of this osteoclastic cell line. On fibronectin, RAW264.7 cells overexpressing periostin moved at an average rate of 1.62 μm/h, whereas control cells moved at 5.85 μm/h (S4b Fig). In the case of untreated dishes, average rate of RAW264.7 cells overexpressing periostin was 1.79 μm/h, and 13.67 μm/h in the case of control RAW264.7 cells (S4b Fig). No conclusive data was obtained on type-1 collagen coated dishes. Interfering periostin expression in this cell line did not showed opposing results as those observed when periostin was overexpressed on adhesion to fibronectin, type-1 collagen or untreated culture dishes (S4c Fig). Taking into account, the inconsistent data on adhesion for the RAW264.7 cell line, and the fact that periostin expression after birth has been detected in osteoblasts on the alveolar bone [44], we decide to use the MC3T3-E1 cell line in the following experiments aimed to describe genes whose expression is altered by periostin overexpression.

### Periostin overexpression influences expression of genes implicated in cell adhesion and migration, and in bone remodelling

**Fig 4:**
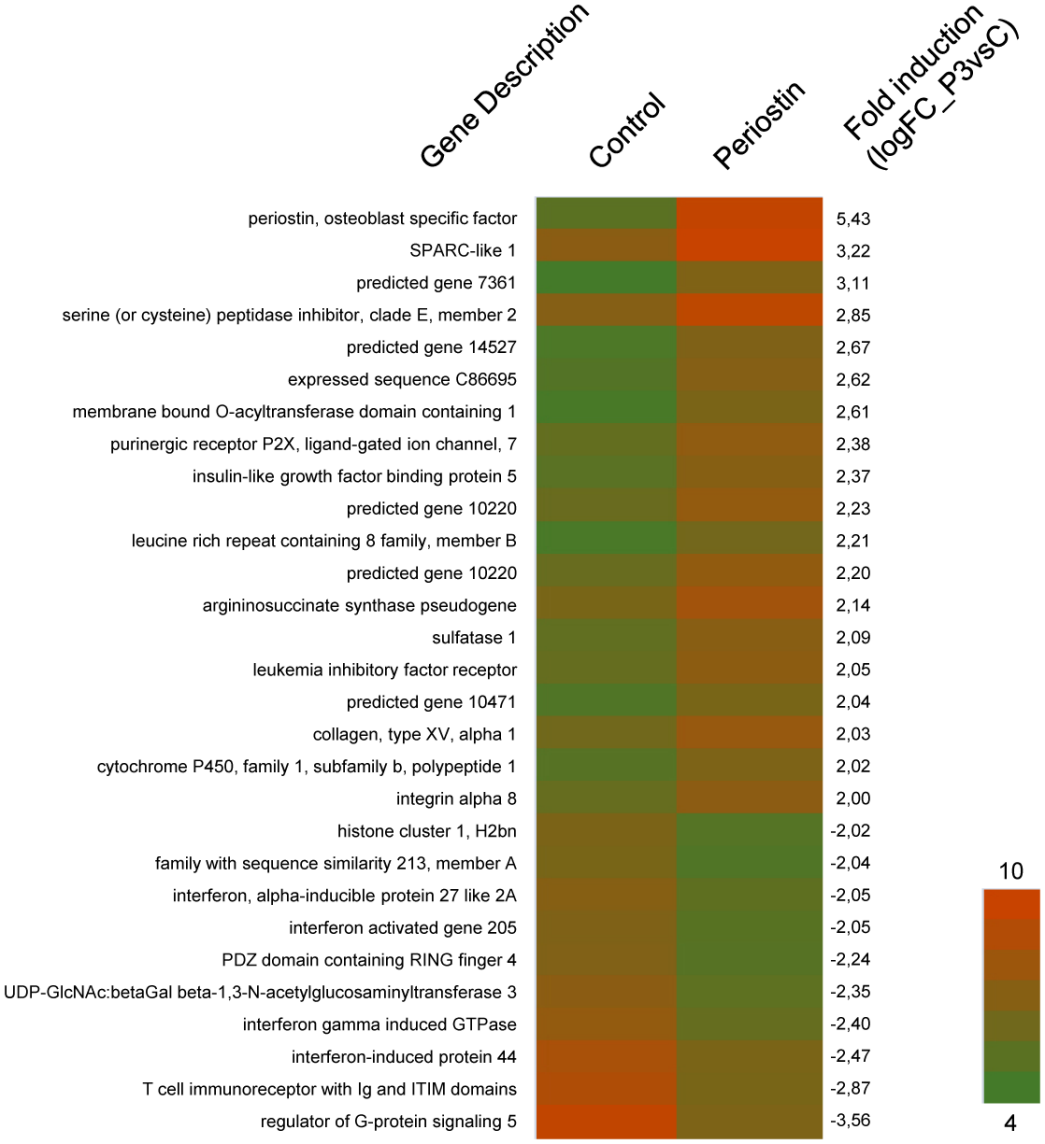
Microarray analysis in MC3T3-E1 periostin overexpressing cells and control MC3T3-E1 cells. Heat map represents the relative expression levels of selected genes (logFC2_P3vsC) as determined by hybridization with GeneChip Mouse Gene 2.0 Array. Left column indicates gene name and Right column indicates logFC_P3vsC value.

It is widely accepted that periostin alters cell behavior in different cell types and in most of the cases it is thought to take place through extracellular matrix remodeling processes. We wanted to know which are the main changes that periostin overexpression produced at the level of gene expression using the MC3T3-E1 clone P3 generated in this work. Gene expression differences between P3 clone and control cells were evaluated by RNA hybridization in a GC Mouse Gene 2.0 Array and using the platform GeneChip^®^ from Affymetrix. The overall results are shown as Supplementary Table 1 (logFC1_P3vsC) and the most important differences can be seen in Fig 4 (logFC2_P3vsC). Of note, Postn, the gene encoding for periostin, is the highest overexpressed gene in clone P3 (logFC_P3vsC = 5.43), followed by Sparcl1 (3.22) and Gm7361 (3.11). By contracts, Rgs5 is the more repressed gene in clone P3 (-3.56), followed by Tigit (-2.87). A preliminary functional analysis of the targets was performed with the list included as S1 Table using the Ingenuity Pathway Analysis platform from Qiagen^®^. A summary with the Top Canonical Pathways and the Top Diseases and Bio Functions of the analyzed data is shown as Table 1. Osteoblasts are known to participate in connective tissue development and especially in skeletal development and function. They are also characterized for being movable and proliferative cells during their physiological function. For what it is reflected in Table 1, periostin overexpression is also able to modify these characteristics by altering gene expression. It is noteworthy that no substantial differences were observed among genes involved in bone differentiation such as alkaline phosphatase (Alpl), runt related transcription factor 2 (Runx2), type-1 collagen alpha 1 (Col1a1), integrin binding sialoprotein (Ibsp) or osteocalcin (Bglap).

**Table 1:** Ingenuity Pathway analysis: data from logFC1_P3vsC (S1 Fig).

Top Canonical Pathways	p-value	Ratio
Hepatic fibrosis/hepatic stellate cell activation	2.04E-08	15/161 (0.093)
Agranulocyte Adhesion and Diapedesis	1.22E-04	9/126 (0.071)
Caveolar-mediated Endocytosis signaling	3.98E-04	6/64 (0.094)
Epithelial adherens junction signaling	3.72E-04	7/131 (0.053)
Acute phase response signaling	4.04E-04	7/133 (0.053
Top Diseases and Bio Functions	p-value	# Molecules
Molecular and Cellular Functions		
Cellular Movement	1,12E-08–5,98E-03	67
Cellular Development	1,14E-08–5,98E-03	86
Cellular Growth and Proliferation	1,14E-08–5,98E-03	86
Cell-To-Cell Signaling and interaction	4,57E-07–5,98E-03	52
Cell Death and Survival	5,98E-06–5,98E-03	85
Physiological System Development and Function		
Skeletal and Muscular System Development and Function	1,12E-08–5,98E-03	54
Connective Tissue Development and Function	1,14E-08–5,98E-03	42
Tissue Development	1,14E-08–5,98E-03	73
Cardiovascular System Development and Function	1,43E-06–5,98E-03	53
Organismal Development	1,43E-06–5,98E-03	72
Diseases and Disordes		
Cancer	2.51E-11–5.98E-03	175
Gastrointestinal Disease	2.51E-11–3.20E-03	112
Metabolic Disease	1.40E-09–4.22E-03	49
Endocrine system Disorders	1.49E-09–1.74E-09	40
Immunological Disease	1.49E-09–5.35E-03	44

**Fig 5:**
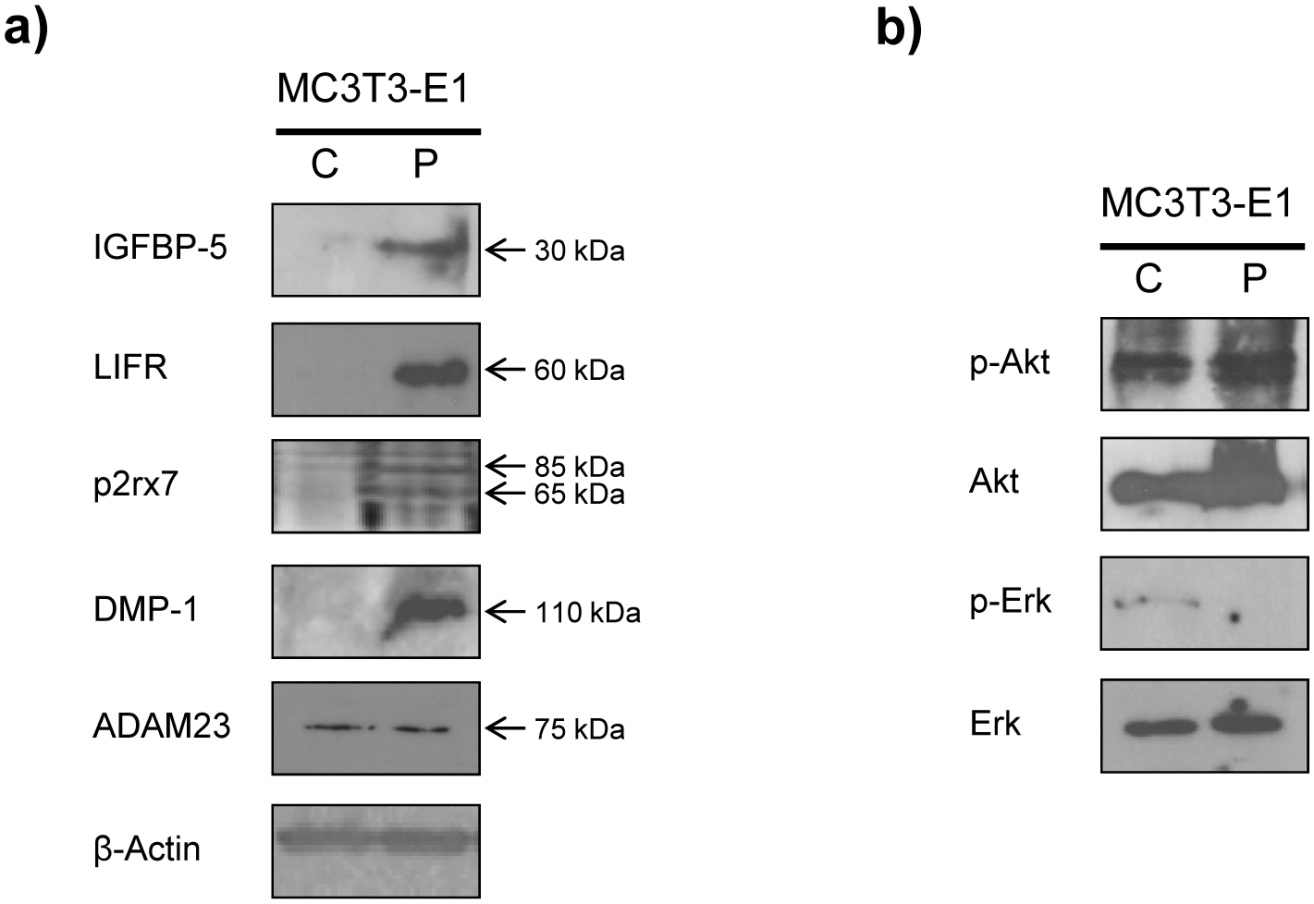
Western-blots detection of proteins whose genes are differentially expressed upon periostin overexpression. a) Proteins detected are indicated on the left and, on the right, molecular weight of detected bands. P2rx7: arrows indicate glycosilated form (top) and unglycosilated form (bottom). LIFR: the truncated form of 60 kDa is shown. b) Levels of p-Akt and p-Erk. C, control cells. P, periostin overexpressing cells.

Following genomic analysis we wanted to examine the effect of these differences at the protein level. To this end, we carried out western-blots analysis using specific antibodies in order to examine if protein expression reflected changes at the genomic level. In this regard, we found that MC3T3-E1 cells overexpressing periostin showed the presence of a immunoreactive band corresponding to the growth factor IGFBP-5 whereas this protein is absent in control cell lines (Fig 5a). Similar results were also observed in the case of the purinergic receptor p2x ligand-gated ion channel p2rx7 where both forms glycosilated and unglycosilated were detected in clone P3 of MC3T3-E1 cells but were absent in the control cells (Fig 5a). Of note, the leukemia inhibitory factor receptor (LIFR) was detected in clone P3 extracts as bands of approximately 60 kDa. However, this band was absent in extracts obtained from control cells (Fig 5a). Apparently, this band corresponds to a non-functional truncated form of the receptor (190 kDa). Protein expression of one of the members of the SIBLING family of integrins, DMP-1, which is important for the correct development of teeth or bones was also examined. DMP-1 protein is present in extracts from periostin-overexpressing cells but absent in extracts obtained from control cells, which correlates with the data obtained at RNA level (S1 Table). No apparent changes in protein levels were detected in the case of ADAM23, a membrane anchored protein involved in cellular adhesion processes.

Periostin downstream effectors after integrin interaction include among others, the Akt/PKB signaling pathway. Activation of Akt and Erk dependent signalling pathways downstream periostin are shown in Fig 5b. The Akt periostin-dependent pathway seems not to be activated by periostin overexpression since both cell extracts, periostin-expressing cells and control cells, showed similar levels of phosphorylated Akt. However, periostin overexpression is able to reduce the phosphorylation levels of Erk in this cell line (Fig 5b).

## Discussion

Matricellular proteins play a pivotal role in tissue homeostasis and in different pathological processes. Among these proteins, periostin is a member of the fascilin-1 family of proteins with known functions in wound repair, bone and teeth morphogenesis and remodeling, oncology, cardiovascular diseases, asthma and in several other inflammatory settings [3, 7, 45]. Periostin importance in bone and teeth metabolism has been underlined after generation of periostin-null mice [11]. In relationship with postnatal teeth development, periostin expression is restricted to the PDL [2, 11], and to the subodontoblast layer [27]. Moreover, it has been also proposed that periostin helps hPDL cells to survive after chronic exposure to periodontal pathogens and proinflammatory cytokines [31]. In all these cases, periostin is able to induce changes in fibroblasts aimed to increase the proliferation rates and to modify migration patterns through interaction with integrins or laminin [26]. In fact, it is generally accepted that periostin increases proliferation and migration rates in different cellular settings in which fibroblasts or fibroblast-like cells are involved [20, 24, 26, 31–32]. In this work we investigated if periostin overexpression could affect cell behavior of osteoblast-like cells. We have observed that the presence of some ECM components stimulates MC3T3-E1 adhesion when periostin is overexpressed. Periostin clearly increases the capacity of this cell line to adhere to type-1 collagen or fibronectin coated surfaces which are known substrates that stimulate MC3T3-E1 cell attachment [41, 46]. Our results clearly indicate that periostin hampers migratory capacities of MC3T3-E1 cells on particular ECM substrates and also in cell culture dishes commonly employed to perform wound healing assays. It has been previously shown that periostin also affects odontoblast proliferation and mineralization [27]. Thus, it could be speculated that periostin functions in bone remodeling process may be different depending on the cell type that express the protein. In this regard and contrary to the enhanced migration induced in fibroblast by periostin, invasion and migration capacities of cancer cells may be also negatively affected by the presence of periostin [33, 47].

Type-1 collagen fibrils are important components in tendon, bone, skin and have been described to co-localized and interact with periostin in the periodontal ligament [8, 44, 48]. Its function is not only important for ECM structure but it is also one of the factors involved in inducing osteoblasts differentiation as well as responsible for the maintenance of the osteoblastic phenotype [49]. Collagen fibrillogenesis occurs at the cell surface and depends on the previous assembly of fibronectin, a large structural protein that forms a bridge between matrix molecules and the cell surface [3, 48, 50]. In this sense, it is not surprising that variations in periostin expression may alter cell behavior towards these extracellular components since periostin is known to increase collagen cross-linking through BMP-1 interaction and lysil oxydase (LOX) activation [6].

Taking advantage of the generation of a periostin overexpressing MC3T3-E1 cell line, we were able to create a list of genes directly influenced by constant endogenous high levels of periostin using RNA array hybridization. It is noteworthy that a considerable number of genes are involved in cell adhesion, migration and bone metabolism. It is important to underline that none of the factors that are involved in positive or negative regulation of periostin expression [45] have shown significant differences in our analysis (logFC_P3vC > 1). For that reason, the main differences in gene expression, and in cellular behavior, may be attributable only to periostin overexpression. Furthermore, some of the genes that have been described to be influenced by periostin expression show no important differences. Thus, sclerostin (sost) mRNA inhibition in bone has been associated with elevated expression of periostin after axial compression [9] whereas our data showed that periostin overexpression in MC3T3-E1 cells barely modifies sost expression (logFC_P3vC = 0.3). Similarly, we did not detect differences in MMP2 expression when MMP2 mRNA and activity has been shown to be induced by periostin [51] either in isolated PDLs or in an experimental orthodontic tooth movement [52]. These aspects strongly suggest that effects due to periostin expression could depend on cell the type and on the existence of certain grade of mechanical stress. MC3T3-E1 cells are known to go into a differentiation process towards an osteogenic phenotype under different stimuli. This process has been previously characterized and several genes have been described as *bona fide* bone formation markers [35, 53–56]. We have looked into our array data for the differences shown for these markers after periostin-overexpression in MC3T3-E1 cells and found no significant differences, i. e. alkaline phosphatase (Alpl)(logFC_P3vC = 0.04), Runx2 (logFC_P3vC = 0.24); Col1a1 (logFC_P3vC = 0.29), sialoprotein (Ibsp)(logFC_P3vC = 0.2), osteocalcin (Bglap)(logFC_P3vC = 0.05). Taking together, these data somehow indicates that this cell line is not following a differentiation process upon periostin overexpression. Thus, the preosteoblastic MC3T3-E1 cell lines created in our work with alterations in periostin expression (i.e. maintenance of high levels of periostin or periostin depletion) could be used as a tool to deepen our knowledge on periostin participation on osteoblastic differentiation. We have observed how periostin affects adhesion profiles and migration properties of this cell line so it is plausible to think that it also might alter the differentiation process under distinct conditions, by using different extracellular matrix components or by exposing cells to mechanical stimuli.

We examined some of the genes altered by periostin overexpression based on their participation on teeth development. Thus, p2rx7 belongs to the family of ATP-gated p2x receptors and null-animals showed phenotypes with diminished inflammatory response, deficiencies in IL-1β release and also skeketal abnormalities [57–58]. P2rx7 might be also involved in astrocyte adhesion by increasing intracellular calcium through a functional link with αvβ3 in a process stimulated by the glycoprotein Thy-1 [59]. IGFBP-5 is one of the intermediates of IGF-1 and IGF-11 actions in many tissues. IGFBP-5 is the most conserved IGFBP across species and was identified as an essential regulator of physiological processes in bone, kidney and mammary gland [60]. In relation with tooth movement, resorption and repair, IGFBP-5 is suggested to be involved in the resorption-repair sequence after orthodontic procedure [61]. Additionally, IGFBP-5 expression has also been implicated in controlling cellular adhesion, cell survival and cell migration in a breast cancer cell line [62]. The LIF receptor (LIFR) belongs to the hematopoietic cytokine receptor family and its ligand (LIF) belongs to the interleukin-6 (IL-6) family of cytokines which are essential for development and life [63]. LIFR downregulation is involved in the induction of cell migration, invasion and metastatic colonization of breast cancer cells [64] and LIFR presence is important for maintenance of pluripontecy of embryonic cells [65]. Interestingly, we detected this receptor as a main band of 60 kDa when the expected size is of about 190 kDa. This 60 kDa band corresponds to a truncated form of the receptor also identified in the differentiation processes towards neuronal lineage or testicular maturation [66–67]. DMP-1 is a known marker of odontoblasts differentiation since it is involved in the mineralization process and in calcium and phosphate metabolism [68–69]. Mechanical loading stimulates expression of both, periostin [9, 70] and DMP-1 [71]. In this work we show that periostin overexpression increases DMP-1 levels. Thus, it can be suggested that one of the mechanisms for DMP-1 induction after mechanical stimuli depends on the presence of periostin. In support of this idea, low levels of DMP-1 are detected in dentin of periostin-deficiente mice [27]. Periostin downstream effectors are mainly derived from periostin interaction with surface proteins. In this sense, periostin binds through its FAS-1 domains to the integrins αvβ3, αvβ5 and α6vβ4, enhancing cell proliferation, survival, migration and metastasis, and involving FAK, Rho/PI3-kinase and Akt/PKB signaling pathways [20, 72–74]. In this sense, we did not observed changes in Akt activation whereas a reduction on Erk activation was clear after periostin overexpression. It is interesting to note that Akt activation can be considered also a marker towards an osteogenic phenotype since Akt phosphorylation is the molecular switch for TGFbeta1-induced osteoblastic differentiation of MC3T3-E1 cells [54]. Erk is an important factor in the regulation of cell migration [75], and reduction of Erk phosphorylation levels may underlie the inhibitory effects on cell migration upon periostin overexpression described in this work. The effect of periostin upon Erk phosphorylation may depend on cell type or cell treatment. For instance, low levels of periostin has been associated to high levels of Erk phosphorylation in lysophosphatidic acid-treated osteosarcoma cells [76].

In summary, in this work we provide new insights into the participation of periostin in the physiology of osteoblasts-like cells. Further work is needed to solve whether periostin is able to directly regulate same effects in vivo under physiological conditions. Meanwhile our data indicate that periostin alters gene expression of very relevant functions related to bone remodeling independently of other stimuli, such as inflammatory cytokines or mechanical stress.

## Supporting Information

S1 Fig MC3T3-E1 periostin-overexpressing clones and adhesion profile of clone P3. (a) Top, western-blot for periostin detection of selected clones. (b) qRT-PCR for periostin expression of selected clones. Bottom, representation of normalized expression using values from Image J densitometry of western-blots. c) Adhesion profile of periostin-overexpressing MC3T3-E1 clone P3. Adhesion profile of MC3T3-E1 periostin-overexpressing clone P3 cells compared to control cells (transfected with and empty vector) to different ECM components. Col I, type I collagen; Col II, type II collagen; Col IV, type IV collagen; FN, fibronectin; LN, laminin; TN, tenascin; VN, vitronectin. Y-axis, relative data refers to fluorometry (485/530nm excitation/emission filters) with blank substraction. Student t test (p < 0.05, *; p < 0.01, **; p < 0.005, ***).
(TIF)

S2 Fig Growth rate of control and P3 clone MC3T3-E1 cells. Estimated growth rates (cell doubling time) were 22.9 h for control cells and 23.8 h for clone P3 cells. Regression value for each cell line is given as R^2^.
(TIF)

S3 Fig Adhesion profiles of MC3T3-E1 periostin shRNA transfected cells. a) Western-blot detecting periostin in cell lysates and conditioned medium from periostin shRNA transfected cells (Sh) compared to MC3T3-E1 cells transfected with an empty vector (C). b) Downregulation of periostin modifies the adhesion profile of MC3T3-E1 cells towards type-1 collagen (COLI), fibronectin (FN) and untreated normal dishes (untreated). Y-axis, relative data refers to fluorometry (485/530nm excitation/emission filters) with blank substraction. Student t test (p < 0.05, *; p < 0.01, **; p < 0.005, ***).
(TIF)

S4 Fig Migration properties of RAW264.7 after periostin overexpression and adhesion profiles of RAW264.7 after periostin shRNA transfection. a) RAW264.7 periostin-overexpressing (P) and control cells (C) were allowed to migrate simultaneously over a 500 μm gap in standard culture dishes commonly employed for wound healing assays (untreated) or wells coated with fibronectin. Pictures at starting (t = 0 h) and final (t = 16h) time points are included. Starting point is indicated with a straight blue line and final points with a dotted line: yellow for RAW264.7 control cells and red for periostin RAW264.7 periostin overexpressing cells. b) Graphical representation of migration rate measured at 16 h from three independent experiments. c) Downregulation of periostin modifies the adhesion profile of RAW264.7s towards fibronectin (FN) but not towards type-1 collagen (COLI) or untreated normal dishes (untreated). Y-axis, relative data refers to fluorometry (485/530nm excitation/emission filters) with blank substraction. Student t test (p < 0.05, *; p < 0.01, **; p < 0.005, ***).
(TIF)

S1 Movie Cell migration on a fibronectin-coated dish. (AVI)

S2 Movie Cell migration on a type-1 collagen-coated dish. (AVI)

S3 Movie Cell migration on an untreated dish. (WMV)

S1 Table Identified genes logFC_P3vC > 1. (PDF)
