# Supplementary figures and images for: Role of Periostin in Adhesion and Migration of Bone Remodeling Cells
Source: PLoS One. 2016 Jan 25;11(1):e0147837. doi: 10.1371/journal.pone.0147837 (PMC4725750; doi:10.1371/journal.pone.0147837)

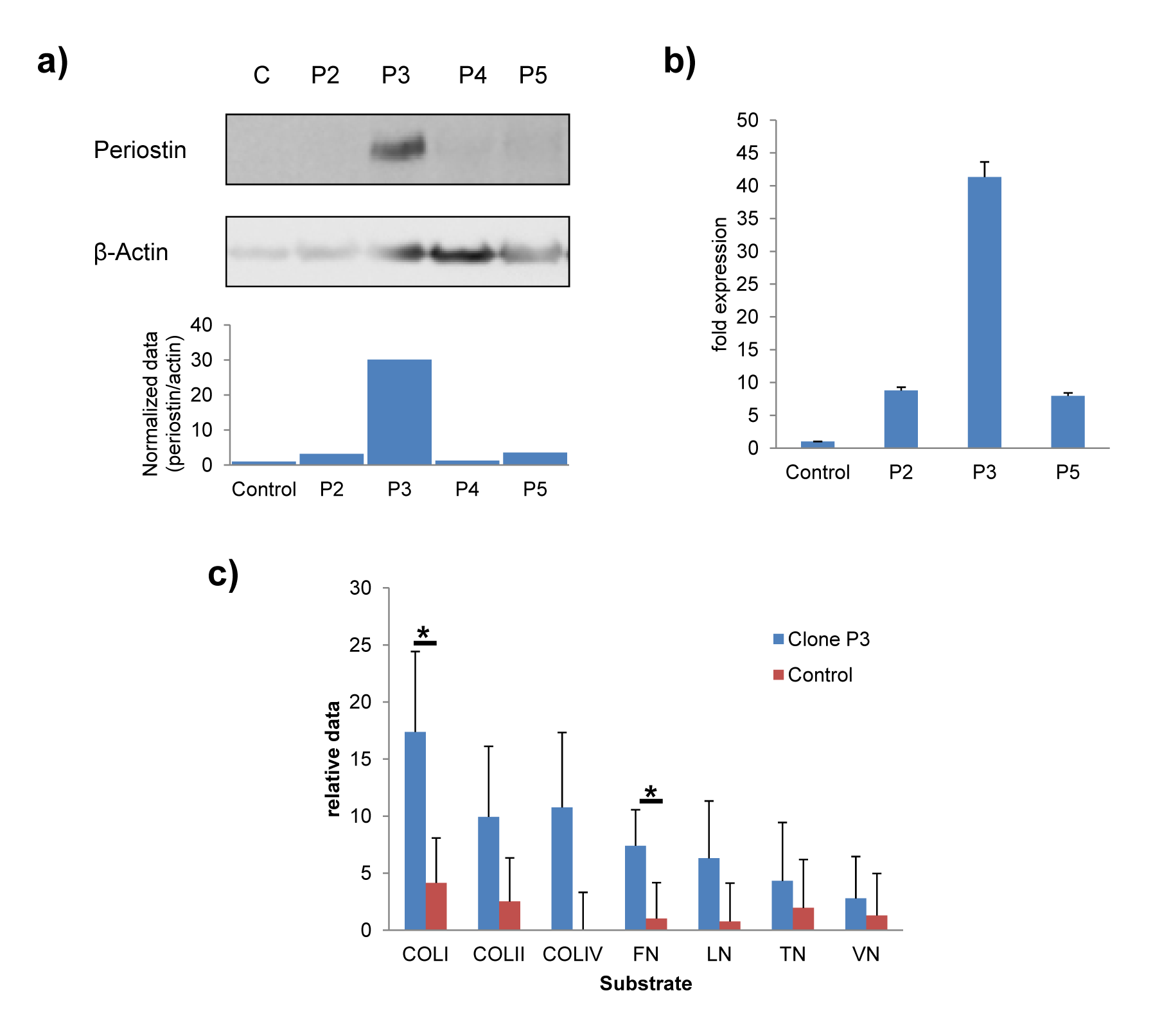

Supplement: S1 Fig — (a) Top, western-blot for periostin detection of selected clones. (b) qRT-PCR for periostin expression of selected clones. Bottom, representation of normalized expression using values from Image J densitometry of western-blots. c) Adhesion profile of periostin-overexpressing MC3T3-E1 clone P3. Adhesion profile of MC3T3-E1 periostin-overexpressing clone P3 cells compared to control cells (transfected with and empty vector) to different ECM components. Col I, type I collagen; Col II, type II collagen; Col IV, type IV collagen; FN, fibronectin; LN, laminin; TN, tenascin; VN, vitronectin. Y-axis, relative data refers to fluorometry (485/530nm excitation/emission filters) with blank substraction. Student t test (p < 0.05, *; p < 0.01, **; p < 0.005, ***). (TIF) [file pone.0147837.s001.tif]

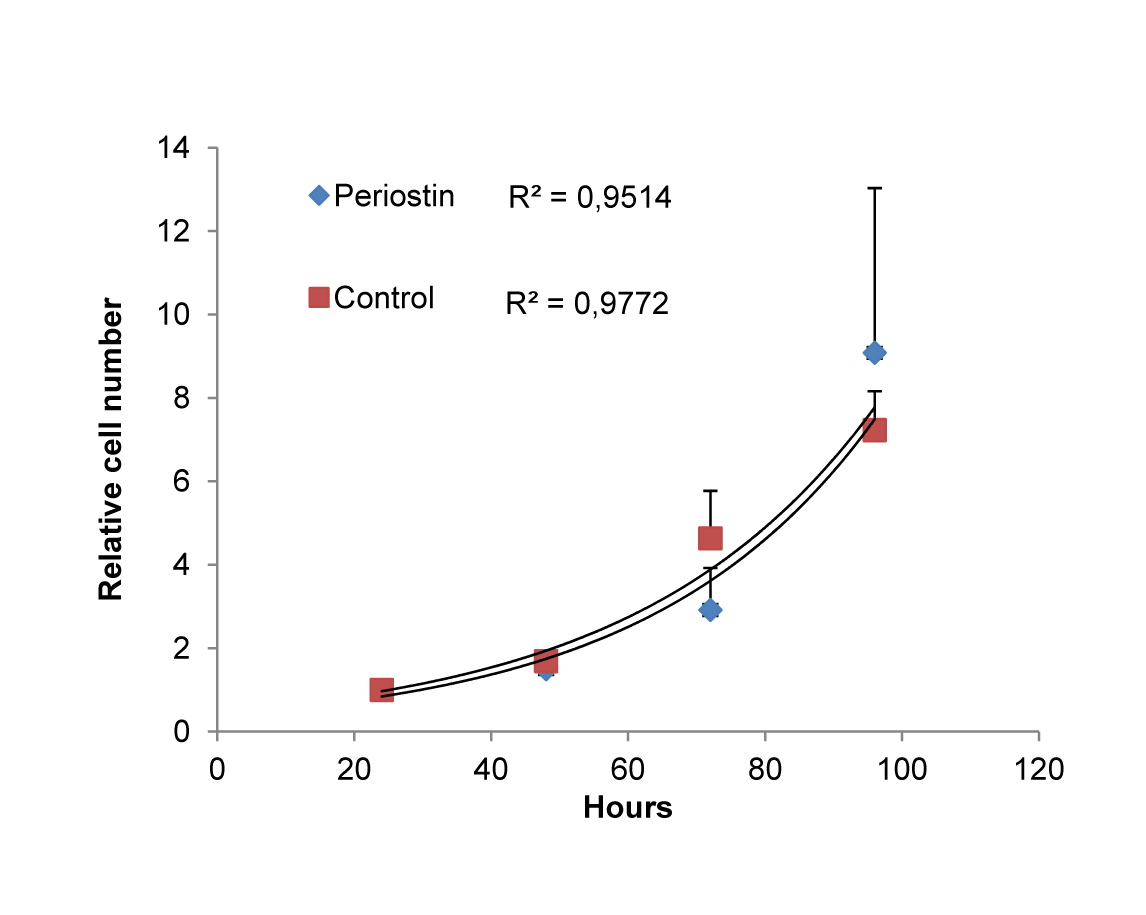

Supplement: S2 Fig — Estimated growth rates (cell doubling time) were 22.9 h for control cells and 23.8 h for clone P3 cells. Regression value for each cell line is given as R2. (TIF) [file pone.0147837.s002.tif]

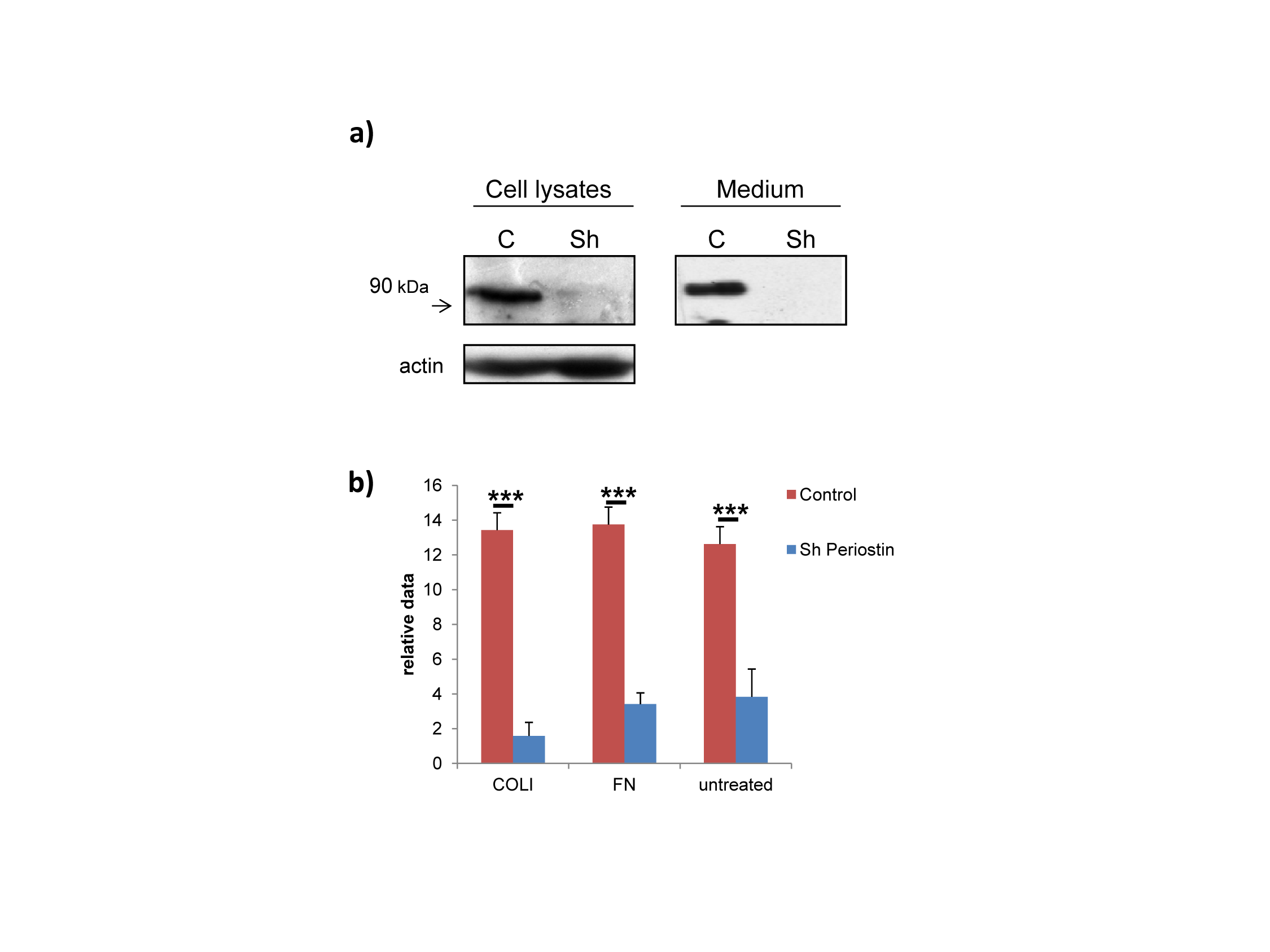

Supplement: S3 Fig — a) Western-blot detecting periostin in cell lysates and conditioned medium from periostin shRNA transfected cells (Sh) compared to MC3T3-E1 cells transfected with an empty vector (C). b) Downregulation of periostin modifies the adhesion profile of MC3T3-E1 cells towards type-1 collagen (COLI), fibronectin (FN) and untreated normal dishes (untreated). Y-axis, relative data refers to fluorometry (485/530nm excitation/emission filters) with blank substraction. Student t test (p < 0.05, *; p < 0.01, **; p < 0.005, ***). (TIF) [file pone.0147837.s003.tif]

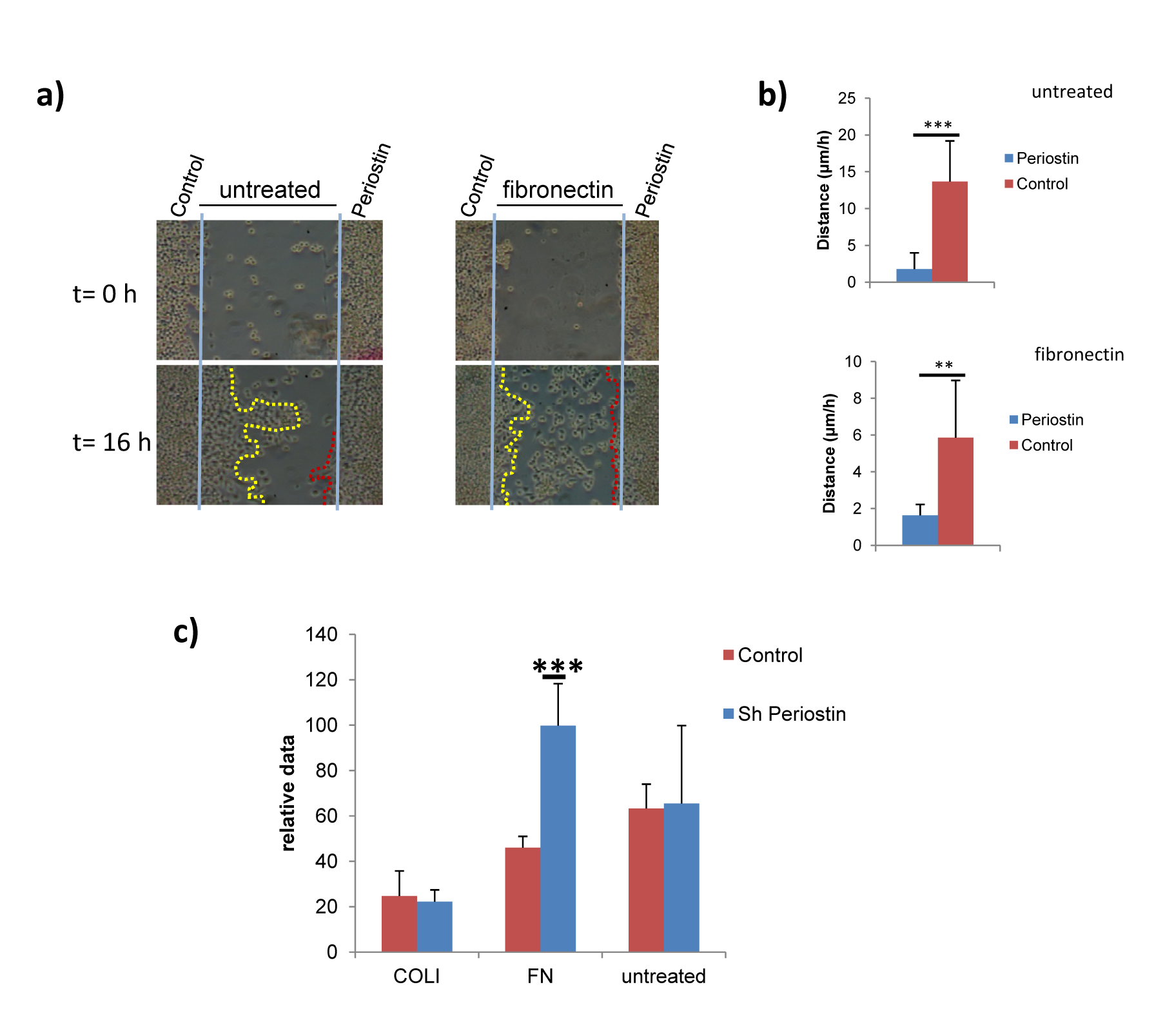

Supplement: S4 Fig — a) RAW264.7 periostin-overexpressing (P) and control cells (C) were allowed to migrate simultaneously over a 500 μm gap in standard culture dishes commonly employed for wound healing assays (untreated) or wells coated with fibronectin. Pictures at starting (t = 0 h) and final (t = 16h) time points are included. Starting point is indicated with a straight blue line and final points with a dotted line: yellow for RAW264.7 control cells and red for periostin RAW264.7 periostin overexpressing cells. b) Graphical representation of migration rate measured at 16 h from three independent experiments. c) Downregulation of periostin modifies the adhesion profile of RAW264.7s towards fibronectin (FN) but not towards type-1 collagen (COLI) or untreated normal dishes (untreated). Y-axis, relative data refers to fluorometry (485/530nm excitation/emission filters) with blank substraction. Student t test (p < 0.05, *; p < 0.01, **; p < 0.005, ***). (TIF) [file pone.0147837.s004.tif]
