# Supplementary material for: Role of Periostin in Adhesion and Migration of Bone Remodeling Cells
Source: PLoS One. 2016 Jan 25;11(1):e0147837. doi: 10.1371/journal.pone.0147837 (PMC4725750; doi:10.1371/journal.pone.0147837)
Supplement: S1 Table — (PDF) [file pone.0147837.s008.pdf]

Supplementary Table 1. Identified genes logFC_P3vC > 1

Probeset	GeneName	GeneDescription	logFC_P3vsC
17397575	Postn	periostin, osteoblast specific factor	5,42592487
17450387	Sparcl1	SPARC-like 1	3,222715686
17435520	Gm7361	predicted gene 7361	3,107949884
17224771	Serpine2	serine (or cysteine) peptidase inhibitor, clade E, member 2	2,84889609
17540741	Gm14527	predicted gene 14527	2,667368944
17499835	C86695	expressed sequence C86695	2,620603141
17286254	Mboat1	membrane bound O-acyltransferase domain containing 1	2,611493761
17446446	NA	NA	2,560534111
17442128	P2rx7	purinergic receptor P2X, ligand-gated ion channel, 7	2,384923323
17224180	Igfbp5	insulin-like growth factor binding protein 5	2,368113779
17446452	Gm10220	predicted gene 10220	2,227925182
17439901	Lrrc8b	leucine rich repeat containing 8 family, member B	2,208338628
17446434	Gm10220	predicted gene 10220	2,196083793
17233769	Gm5424	argininosuccinate synthase pseudogene	2,135988641
17211198	Sulf1	sulfatase 1	2,091447763
17310044	Lifr	leukemia inhibitory factor receptor	2,052237776
17446499	Gm10471	predicted gene 10471	2,04394507
17413866	Col15a1	collagen, type XV, alpha 1	2,029870997
17347448	Cyp1b1	cytochrome P450, family 1, subfamily b, polypeptide 1	2,017567309
17381717	Itga8	integrin alpha 8	2,003049116
17227077	Prelp	proline arginine-rich end leucine-rich repeat	1,990489278
17213608	Adam23	a disintegrin and metallopeptidase domain 23	1,960873702
17468183	Actg2	actin, gamma 2, smooth muscle, enteric	1,899430078
17479769	Adamtsl3	ADAMTS-like 3	1,859255652
17462149	Cxcl12	chemokine (C-X-C motif) ligand 12	1,841571256
17446482	Speer4a	spermatogenesis associated glutamate (E)-rich protein 4a	1,831976399
17355607	Setbp1	SET binding protein 1	1,794853784
17500832	Fat1	FAT tumor suppressor homolog 1 (Drosophila)	1,767811408
17221536	Defb41	defensin beta 41	1,766731584
17446060	AI506816	expressed sequence AI506816	1,760732541
17446466	Gm5862	predicted gene 5862	1,758775357
17316793	Rspo2	R-spondin 2 homolog (Xenopus laevis)	1,755164822
17507377	Col4a1	collagen, type IV, alpha 1	1,740439736
17510261	Gm17576	predicted gene, 17576	1,726943408
17466033	Hipk2	homeodomain interacting protein kinase 2	1,701027432
17282570	Ltbp2	latent transforming growth factor beta binding protein 2	1,693022591
17398115	Ptx3	pentraxin related gene	1,680776932
17548238	Gm3601	predicted gene 3601	1,679907522
17492947	9930013L23Rik	RIKEN cDNA 9930013L23 gene	1,666096799
17439805	Dmp1	dentin matrix protein 1	1,658799905
17548717	Gm3601	predicted gene 3601	1,658769754
17438963	Ppbp	pro-platelet basic protein	1,645451884
17214729	Serpine2	serine (or cysteine) peptidase inhibitor, clade E, member 2	1,645279182
17395129	Mir296	microRNA 296	1,637718145
17510136	Ifi30	interferon gamma inducible protein 30	1,634597847
17519649	Gsta4	glutathione S-transferase, alpha 4	1,634066652
17248691	Ebf1	early B cell factor 1	1,602953551
17480880	Pde2a	phosphodiesterase 2A, cGMP-stimulated	1,595577707
17471222	Ccnd2	cyclin D2	1,586624606
17374618	Disp2	dispatched homolog 2 (Drosophila)	1,566338189
17224661	Epha4	Eph receptor A4	1,542445406
17291638	1700018A04Rik	RIKEN cDNA 1700018A04 gene	1,513721163
17498962	Col4a2	collagen, type IV, alpha 2	1,513099516
17230830	Tgfb2	transforming growth factor, beta 2	1,504999136
17331078	Tmem45a	transmembrane protein 45a	1,45364587
17545106	Capn6	calpain 6	1,453518959
17400599	Txnip	thioredoxin interacting protein	1,427931706
17508609	Nrg1	neuregulin 1	1,415518318
17310661	March11	membrane-associated ring finger (C3HC4) 11	1,401681688
17545824	Nhs	Nance-Horan syndrome (human)	1,398600468
17315743	Osmr	oncostatin M receptor	1,394349047
17377144	Slc24a3	solute carrier family 24 (sodium/potassium/calcium exchanger), membe	1,390704293

17228667	Pappa2	pappalysin 2	1,384336062
17495260	Gm5600	predicted gene 5600	1,382560342
17316043	Npr3	natriuretic peptide receptor 3	1,377771317
17310770	NA	NA	1,374590093
17248470	NA	NA	1,374579047
17470681	NA	NA	1,347554157
17508132	Mir3107	microRNA 3107	1,347217454
17514333	Gm10719	predicted gene 10719	1,345235705
17403743	NA	NA	1,343461548
17376822	Snap25	synaptosomal-associated protein 25	1,342230896
17216458	Serpinb2	serine (or cysteine) peptidase inhibitor, clade B, member 2	1,339904866
17486096	Olfr1349	olfactory receptor 1349	1,335213436
17337133	H2-Q7	histocompatibility 2, Q region locus 7	1,330642585
17421540	Nppb	natriuretic peptide type B	1,328679216
17212696	Tmeff2	transmembrane protein with EGF-like and two follistatin-like domains 2	1,32691956
17316197	Fam105a	family with sequence similarity 105, member A	1,322609575
17502521	F2rl3	coagulation factor II (thrombin) receptor-like 3	1,300108904
17389467	Grem1	gremlin 1	1,294759625
17514349	Gm10106	predicted gene 10106	1,293155073
17281451	NA	NA	1,291755706
17289811	NA	NA	1,289819992
17469136	Adamts9	a disintegrin-like and metallopeptidase (reprolysin type) with thrombospo	1,285276303
17491730	A330076H08Rik	RIKEN cDNA A330076H08 gene	1,275729485
17311519	Nov	nephroblastoma overexpressed gene	1,267482269
17445651	Gm10354	predicted gene 10354	1,266398415
17446440	Gm21671	predicted gene, 21671	1,25467057
17330156	NA	NA	1,249567333
17288239	Gm10324	predicted gene 10324	1,247529852
17283380	Fbln5	fibulin 5	1,246804862
17411262	St6galnac3	ST6 (alpha-N-acetyl-neuraminyl-2,3-beta-galactosyl-1,3)-N-acetylgalacto	1,242011474
17334782	Fbxl16	F-box and leucine-rich repeat protein 16	1,236947373
17508591	1810029E06Rik	RIKEN cDNA 1810029E06 gene	1,236129964
17376723	Gm14074	predicted gene 14074	1,235731577
17302429	Lmo7	LIM domain only 7	1,228958863
17545488	Kctd12b	potassium channel tetramerisation domain containing 12b	1,228657477
17384175	NA	NA	1,228311561
17211305	Pi15	peptidase inhibitor 15	1,220843527
17231694	Plagl1	pleiomorphic adenoma gene-like 1	1,219920888
17412913	Il11ra1	interleukin 11 receptor, alpha chain 1	1,219680524
17222332	Mgat4a	mannoside acetylglucosaminyltransferase 4, isoenzyme A	1,214943507
17404073	Fabp5	fatty acid binding protein 5, epidermal	1,210195215
17510114	Gdf15	growth differentiation factor 15	1,207378873
17509752	Gm15992	predicted gene 15992	1,202660874
17236288	Igf1	insulin-like growth factor 1	1,200839114
17491741	NA	NA	1,199218642
17234705	Mir1930	microRNA 1930	1,197063483
17226313	Steap3	STEAP family member 3	1,19044731
17277679	Gm2022	predicted pseudogene 2022	1,189035944
17317046	Tnfrsf11b	tumor necrosis factor receptor superfamily, member 11b (osteoprotegeri	1,18148243
17305163	2610528A11Rik	RIKEN cDNA 2610528A11 gene	1,180512152
17300027	NA	NA	1,168681593
17501810	Nr2c2ap	nuclear receptor 2C2-associated protein	1,160869774
17378663	Myl9	myosin, light polypeptide 9, regulatory	1,159371502
17338872	Ndufa11	NADH dehydrogenase (ubiquinone) 1 alpha subcomplex 11	1,156181789
17514326	Gm10722	predicted gene 10722	1,155929282
17232235	Ctgf	connective tissue growth factor	1,155598003
17437227	Gm16401	predicted gene 16401	1,151424627
17523676	NA	NA	1,14678757
17364098	Acta2	actin, alpha 2, smooth muscle, aorta	1,145348416
17385710	NA	NA	1,145223663
17474820	Nlrp5	NLR family, pyrin domain containing 5	1,138989556
17501940	Crlf1	cytokine receptor-like factor 1	1,134853699
17510057	Klhl26	kelch-like 26	1,133513884
17487666	Vmn1r175	vomeronasal 1 receptor 175	1,128044219
17502001	Jund	Jun proto-oncogene related gene d	1,124990624
17464706	Dlx5	distal-less homeobox 5	1,123905371
17416212	1700024P16Rik	RIKEN cDNA 1700024P16 gene	1,115131413

17518191	Itga11	integrin alpha 11	1,105148354
17544623	NA	NA	1,09918175
17216751	NA	NA	1,096516335
17501217	NA	NA	1,095981784
17295136	Iqgap2	IQ motif containing GTPase activating protein 2	1,094584396
17453819	Serpine1	serine (or cysteine) peptidase inhibitor, clade E, member 1	1,092325091
17518563	Rasl12	RAS-like, family 12	1,090010793
17509907	Gatad2a	GATA zinc finger domain containing 2A	1,086973881
17298956	Mat1a	methionine adenosyltransferase I, alpha	1,081040417
17301414	Scara5	scavenger receptor class A, member 5 (putative)	1,078155347
17538171	Mid2	midline 2	1,076899829
17462841	NA	NA	1,076046345
17224146	March4	membrane-associated ring finger (C3HC4) 4	1,075014417
17501994	Lsm4	LSM4 homolog, U6 small nuclear RNA associated (S. cerevisiae)	1,074850116
17381589	Itih2	inter-alpha trypsin inhibitor, heavy chain 2	1,0736663
17244705	Acss3	acyl-CoA synthetase short-chain family member 3	1,071691977
17217291	Plekha6	pleckstrin homology domain containing, family A member 6	1,067304159
17248664	Ublcp1	ubiquitin-like domain containing CTD phosphatase 1	1,066917196
17439909	Lrrc8c	leucine rich repeat containing 8 family, member C	1,066718482
17269156	Gm11937	predicted gene 11937	1,055307144
17433328	Per3	period circadian clock 3	1,055307144
17431216	Catsper4	cation channel, sperm associated 4	1,054391002
17266196	Ccdc55	coiled-coil domain containing 55	1,053908184
17530406	Acpp	acid phosphatase, prostate	1,04525308
17234146	Fam13c	family with sequence similarity 13, member C	1,044412669
17251220	NA	NA	1,043399259
17485826	Ptprh	protein tyrosine phosphatase, receptor type, H	1,040727136
17498847	Cers4	ceramide synthase 4	1,037167136
17304336	NA	NA	1,035472439
17214166	Pnkd	paroxysmal nonkinesiogenic dyskinesia	1,035286202
17401902	Col11a1	collagen, type XI, alpha 1	1,033919015
17288112	Mir24-1	microRNA 24-1	1,033633045
17278696	Mir665	microRNA 665	1,033119712
17469656	Srgap3	SLIT-ROBO Rho GTPase activating protein 3	1,032670583
17504190	Gpr56	G protein-coupled receptor 56	1,028469339
17229178	Atp1b1	ATPase, Na+/K+ transporting, beta 1 polypeptide	1,025578177
17510120	Pgpep1	pyroglutamyl-peptidase I	1,023285319
17223811	NA	NA	1,023180318
17232281	NA	NA	1,022552989
17510072	2810428I15Rik	RIKEN cDNA 2810428I15 gene	1,022502951
17501891	Cope	coatomer protein complex, subunit epsilon	1,021768216
17502573	Hmox1	heme oxygenase (decycling) 1	1,019037235
17317153	Sntb1	syntrophin, basic 1	1,014748952
17471087	Tuba3a	tubulin, alpha 3A	1,010269336
17434890	Cacna2d1	calcium channel, voltage-dependent, alpha2/delta subunit 1	1,010134759
17446507	Gm10220	predicted gene 10220	1,009055358
17213578	NA	NA	1,006692762
17213246	Cdk15	cyclin-dependent kinase 15	1,005941203
17520254	Adamts7	a disintegrin-like and metallopeptidase (reprolysin type) with thrombospo	1,004563076
17348396	NA	NA	1,004563076
17450944	NA	NA	1,004231731
17460569	Gp9	glycoprotein 9 (platelet)	1,004231731
17221375	Eya1	eyes absent 1 homolog (Drosophila)	1,002096975
17501950	Fkbp8	FK506 binding protein 8	1,001112557
17292069	Smim13	small integral membrane protein 13	-1,000993295
17463387	Tspan11	tetraspanin 11	-1,00113049
17404601	Gnb4	guanine nucleotide binding protein (G protein), beta 4	-1,003383887
17294734	NA	NA	-1,004231731
17521541	Uba7	ubiquitin-like modifier activating enzyme 7	-1,006230807
17256565	Tubg2	tubulin, gamma 2	-1,006242009
17376274	Nop56	NOP56 ribonucleoprotein	-1,006593192
17371390	Nostrin	nitric oxide synthase trafficker	-1,006878975
17246033	Gm16230	predicted gene 16230	-1,008785904
17432770	Gm13157	predicted gene 13157	-1,008785904
17292327	Mirlet7f-1	microRNA let7f-1	-1,010731919
17214981	NA	NA	-1,011754173
17250567	Aldh3a1	aldehyde dehydrogenase family 3, subfamily A1	-1,013433707

17450461	Gbp4	guanylate binding protein 4	-1,013979003
17281684	NA	NA	-1,015288637
17350916	Gm4951	predicted gene 4951	-1,020025698
17312686	Apol9b	apolipoprotein L 9b	-1,022726288
17280270	Gm9292	predicted gene 9292	-1,02434272
17411319	Rabggtb	RAB geranylgeranyl transferase, b subunit	-1,027591029
17434023	Isg15	ISG15 ubiquitin-like modifier	-1,027833478
17538562	Gm15091	predicted gene 15091	-1,02883387
17266368	Snord42a	small nucleolar RNA, C/D box 42A	-1,030191846
17387385	Calcrl	calcitonin receptor-like	-1,031253613
17249801	Gm2a	GM2 ganglioside activator protein	-1,033096419
17227266	Elf3	E74-like factor 3	-1,033633045
17263031	Snord1c	small nucleolar RNA, C/D box 1C	-1,033982086
17326180	Gm19723	predicted gene, 19723	-1,035472439
17537853	Bhlhb9	basic helix-loop-helix domain containing, class B9	-1,03734823
17501440	Ddx60	DEAD (Asp-Glu-Ala-Asp) box polypeptide 60	-1,041606122
17348879	Asxl3	additional sex combs like 3 (Drosophila)	-1,041797896
17513672	Cyba	cytochrome b-245, alpha polypeptide	-1,042910703
17470796	Ptpn6	protein tyrosine phosphatase, non-receptor type 6	-1,043322727
17262428	Zfp2	zinc finger protein 2	-1,044421332
17285677	Hist1h2ai	histone cluster 1, H2ai	-1,045665333
17372894	Olfr1158	olfactory receptor 1158	-1,050391988
17277788	Flrt2	fibronectin leucine rich transmembrane protein 2	-1,050414596
17379113	C330008G21Rik	RIKEN cDNA C330008G21 gene	-1,055543667
17277170	Acot6	acyl-CoA thioesterase 6	-1,057242063
17541008	Snora69	small nucleolar RNA, H/ACA box 69	-1,057512152
17252847	NA	NA	-1,060187053
17248809	Havcr2	hepatitis A virus cellular receptor 2	-1,060549786
17547604	Lgals3bp	lectin, galactoside-binding, soluble, 3 binding protein	-1,060557157
17547684	NA	NA	-1,061990785
17287243	NA	NA	-1,065363485
17258638	Snord1b	small nucleolar RNA, C/D box 1B	-1,065492345
17499792	Defa17	defensin, alpha, 17	-1,06608693
17313050	Apobec3	apolipoprotein B mRNA editing enzyme, catalytic polypeptide 3	-1,071244959
17312052	NA	NA	-1,077782455
17463909	Mgst1	microsomal glutathione S-transferase 1	-1,081316416
17493005	Fah	fumarylacetoacetate hydrolase	-1,087149479
17256734	Ifi35	interferon-induced protein 35	-1,087485983
17331543	Samsn1	SAM domain, SH3 domain and nuclear localization signals, 1	-1,093082682
17533812	E330010L02Rik	RIKEN cDNA E330010L02 gene	-1,093665898
17330119	Dtx3l	deltex 3-like (Drosophila)	-1,094997172
17403224	Gbp7	guanylate binding protein 7	-1,105669964
17240621	Aim1	absent in melanoma 1	-1,10720769
17214921	Gm16094	predicted gene 16094	-1,114577948
17481693	1600010M07Rik	RIKEN cDNA 1600010M07 gene	-1,122980827
17399347	Thbs3	thrombospondin 3	-1,123376984
17503360	NA	NA	-1,125772078
17500620	AI429214	expressed sequence AI429214	-1,127600001
17548266	Actl6a	actin-like 6A	-1,134931228
17272785	Lgals3bp	lectin, galactoside-binding, soluble, 3 binding protein	-1,1361988
17239040	Ulbp1	UL16 binding protein 1	-1,138904241
17517573	NA	NA	-1,148845667
17230005	Gm4955	predicted gene 4955	-1,149859309
17267629	Gm11496	predicted gene 11496	-1,150803205
17238822	NA	NA	-1,155771544
17383672	Gm14488	predicted gene 14488	-1,158327315
17269464	Jup	junction plakoglobin	-1,158590165
17303706	Gng2	guanine nucleotide binding protein (G protein), gamma 2	-1,175306901
17247176	Ramp3	receptor (calcitonin) activity modifying protein 3	-1,178102742
17448821	Ociad2	OCIA domain containing 2	-1,188293851
17376441	Gm11037	predicted gene 11037	-1,194797089
17496947	Fgfr2	fibroblast growth factor receptor 2	-1,197101318
17394478	2810408M09Rik	RIKEN cDNA 2810408M09 gene	-1,206319826
17546935	Rbmy	RNA binding motif protein, Y chromosome	-1,207134008
17546944	Rbmy	RNA binding motif protein, Y chromosome	-1,207134008
17546345	Rbmy	RNA binding motif protein, Y chromosome	-1,209468881
17546953	Rbmy	RNA binding motif protein, Y chromosome	-1,209468881

17306906	Ripk3	receptor-interacting serine-threonine kinase 3	-1,212920513
17254047	Ccl7	chemokine (C-C motif) ligand 7	-1,216428112
17336446	Psmb8	proteasome (prosome, macropain) subunit, beta type 8 (large multifunct	-1,216798959
17278290	Serpina3g	serine (or cysteine) peptidase inhibitor, clade A, member 3G	-1,218053573
17293982	Zfp874b	zinc finger protein 874b	-1,219358119
17482764	NA	NA	-1,229792972
17285834	Hist1h2bg	histone cluster 1, H2bg	-1,234358263
17291208	Hist1h4m	histone cluster 1, H4m	-1,235475592
17358832	Ifit1	interferon-induced protein with tetratricopeptide repeats 1	-1,24140537
17344120	Snord52	small nucleolar RNA, C/D box 52	-1,250673298
17445627	NA	NA	-1,253259351
17337816	Cyp39a1	cytochrome P450, family 39, subfamily a, polypeptide 1	-1,271093734
17322163	Itgb7	integrin beta 7	-1,280723576
17305278	Gm3676	predicted gene 3676	-1,299038673
17268353	Gm11517	ubiquitin A-52 residue ribosomal protein fusion product 1 pseudogene	-1,310350025
17395376	LOC100045326	zinc finger protein 120-like	-1,311852112
17300169	Traj41	T cell receptor alpha joining 41	-1,313437915
17265748	Aspa	aspartoacylase	-1,317388335
17330099	Parp14	poly (ADP-ribose) polymerase family, member 14	-1,321398961
17546963	Rbmy	RNA binding motif protein, Y chromosome	-1,328359567
17430928	NA	NA	-1,337024698
17544696	Nxf3	nuclear RNA export factor 3	-1,376873134
17240880	Man1a	mannosidase 1, alpha	-1,378986454
17423395	Slc26a7	solute carrier family 26, member 7	-1,379965726
17305372	Gm17079	predicted gene 17079	-1,385805517
17407435	Lce1b	late cornified envelope 1B	-1,394343363
17507306	NA	NA	-1,399863886
17257937	Kcnj2	potassium inwardly-rectifying channel, subfamily J, member 2	-1,399863886
17300067	Trav7-6	T cell receptor alpha variable 7-6	-1,400549639
17380392	Gm14444	predicted gene 14444	-1,403940142
17481960	Arntl	aryl hydrocarbon receptor nuclear translocator-like	-1,406271545
17401269	Ptpn22	protein tyrosine phosphatase, non-receptor type 22 (lymphoid)	-1,426351458
17546333	Rbmy	RNA binding motif protein, Y chromosome	-1,444886393
17546355	Rbmy	RNA binding motif protein, Y chromosome	-1,444886393
17285821	Hist1h3g	histone cluster 1, H3g	-1,446334999
17517105	Il18	interleukin 18	-1,450488606
17285846	Hist1h4m	histone cluster 1, H4m	-1,462670703
17235582	Snord37	small nucleolar RNA, C/D box 37	-1,470006892
17284936	Akr1c13	aldo-keto reductase family 1, member C13	-1,471029306
17291222	Hist1h3c	histone cluster 1, H3c	-1,478681308
17478789	Ndn	necdin	-1,48372336
17316780	Angpt1	angiopoietin 1	-1,488190895
17414059	NA	NA	-1,495311119
17291233	Hist1h3a	histone cluster 1, H3a	-1,511268249
17508833	NA	NA	-1,512070062
17408021	Hist2h4	histone cluster 2, H4	-1,531532643
17456963	Mir335	microRNA 335	-1,538681952
17475818	AF357399	snoRNA AF357399	-1,551438327
17332531	Mx1	myxovirus (influenza virus) resistance 1	-1,552266179
17291180	Hist1h4m	histone cluster 1, H4m	-1,555958324
17546923	Rbmy	RNA binding motif protein, Y chromosome	-1,558836907
17541053	Rhox4f	reproductive homeobox 4F	-1,56378337
17403237	Gbp3	guanylate binding protein 3	-1,598600488
17537861	Arxes2	adipocyte-related X-chromosome expressed sequence 2	-1,600973661
17288780	Mctp1	multiple C2 domains, transmembrane 1	-1,609884324
17450142	Hpse	heparanase	-1,626652907
17230087	Ifi203	interferon activated gene 203	-1,64474556
17487507	n-R5s152	nuclear encoded rRNA 5S 152	-1,660335185
17378827	Lbp	lipopolysaccharide binding protein	-1,675094042
17441037	Oasl2	2'-5' oligoadenylate synthetase-like 2	-1,691553223
17486549	Vmn2r55	vomeronasal 2, receptor 55	-1,724182448
17249990	Irgm2	immunity-related GTPase family M member 2	-1,726050498
17320967	Amigo2	adhesion molecule with Ig like domain 2	-1,727323659
17285815	Hist1h4h	histone cluster 1, H4h	-1,746244838
17324446	Rtp4	receptor transporter protein 4	-1,757997317
17533665	Usp11	ubiquitin specific peptidase 11	-1,774795566
17300591	Irf9	interferon regulatory factor 9	-1,778793956

17274620	Cmpk2	cytidine monophosphate (UMP-CMP) kinase 2, mitochondrial	-1,828358635
17266946	Ccl5	chemokine (C-C motif) ligand 5	-1,846500456
17385797	Ifih1	interferon induced with helicase C domain 1	-1,857725306
17230045	Ifi204	interferon activated gene 204	-1,957188156
17285746	Hist1h2bn	histone cluster 1, H2bn	-2,018288344
17417115	NA	NA	-2,029569463
17305221	Fam213a	family with sequence similarity 213, member A	-2,040375446
17283549	Ifi27l2a	interferon, alpha-inducible protein 27 like 2A	-2,046783145
17230111	Ifi205	interferon activated gene 205	-2,049658575
17314421	Pdzrn4	PDZ domain containing RING finger 4	-2,244869219
17510422	B3gnt3	UDP-GlcNAc:betaGal beta-1,3-N-acetylglucosaminyltransferase 3	-2,354990029
17249980	Igtp	interferon gamma induced GTPase	-2,400328757
17411147	Ifi44	interferon-induced protein 44	-2,466292466
17330478	Tigit	T cell immunoreceptor with Ig and ITIM domains	-2,866748124
17229451	Rgs5	regulator of G-protein signaling 5	-3,532173796
17219139	Rgs5	regulator of G-protein signaling 5	-3,561233047
